# Is Initial Misdiagnosis Associated with Reaching Disability Milestones in Patients with Multiple Sclerosis?

**DOI:** 10.3390/medicina56040170

**Published:** 2020-04-10

**Authors:** Alina Ivaniuk, Tetiana Marusich, Yuliia Solodovnikova, Anatoliy Son

**Affiliations:** Department of Neurology and Neurosurgery, Odessa National Medical University, 65082 Odessa, Ukraine; tatyanasav8@ukr.net (T.M.); julie-sinel@ukr.net (Y.S.); neuroson@ukr.net (A.S.)

**Keywords:** multiple sclerosis, misdiagnosis, prognosis, disability, EDSS

## Abstract

*Background and objectives*: multiple sclerosis (MS) is a chronic demyelinating disorder of the CNS with a variable course and disability progression. The latter may be prevented with disease-modifying therapy (DMT). Initial misdiagnosis may postpone the use of DMT. There are no studies to explore whether initial misdiagnosis is indeed associated with a higher rate of reaching disability in MS patients. We aimed to investigate the association between initial misdiagnosis and reaching disability milestones in relapsing-remitting MS (RR-MS) patients. *Materials and methods*: Data from 128 RR-MS patients were retrospectively reviewed. EDSS 4 and EDSS 6 were chosen as disability milestones as those associated with a significant decrease in ambulation. Survival analysis was used, and Kaplan–Meier curves were generated to investigate how initial misdiagnosis affects reaching the defined milestones. *Results*: 53 patients (41.4%, 31 females, 22 males) were initially misdiagnosed. Initially misdiagnosed patients had a lesser risk of reaching EDSS 4 up to 11 years and EDSS 6 up to 22 years from the onset than non-misdiagnosed patients (*p* = 0.22 and *p* = 0.25 correspondingly). Median time to reaching EDSS 4 and 6 was eight years (95% CI 0.0–17.6) and 10 years (95% CI 4.25–20.75) in misdiagnosed and three years (95% CI 0.0–20.0 years) and five years (95% CI 0.0–13.73 years) in non-misdiagnosed patients correspondingly. Conclusions: Initially misdiagnosed RR-MS patients tended to reach disability milestones later than non-misdiagnosed ones, which might reflect an intrinsically milder disease. Individuals presenting with mild or non-specific symptoms suspicious of MS, must be deliberately managed.

## 1. Introduction

Multiple sclerosis (MS) is a chronic demyelinating immune-mediated disease of the CNS. Its presentation varies based on the CNS sites affected by demyelination. It can include sensory, motor, visual and/or balance disturbances, impairment of bowel and/or bladder function, disrupted emotions and cognition, etc. Global MS prevalence is estimated to be 50–300 per 100,000, with 2–3 million people affected worldwide [1]. MS causes a substantial social impact and economic burden since the incidence of the disease peaks at the mid-thirties (i.e., productive population), and it became the world-leading cause of non-traumatic neurological disability among young people [2]. Relapsing-remitting multiple sclerosis (RR-MS) is the most common MS phenotype accounting for 85%–90% of MS cases [3,4]. In RR-MS, accumulation of disability displays a high variability and depends on the number and severity of relapses. Eventually, people affected with RR-MS who either do not receive disease-modifying therapy (DMT) or fail to achieve relapse rate control develop an irreversible disability. New DMT agents are developed aiming to achieve a better therapeutic potential and a more favorable efficacy-safety profile. Early DMT initiation can result in a decrease in the proportion of people who reach significant disability or at least considerably slow down the functional decline, although the long-term efficacy is still debatable [5].

McDonalds criteria are the criteria for MS diagnosis, which were introduced in 2001 and underwent several editions to their current 2017 version [4]. Despite the well-validated criteria, MS can still be misdiagnosed. It may take years, since the first referral to the healthcare professional to the establishment of a correct diagnosis. While other disorders are more often initially diagnosed as MS in high-income countries [6], the opposite problem may be particularly relevant to low-income countries, especially for rural sites with limited access to healthcare professionals and corresponding diagnostic procedures.

Patients and their relatives, as well as doctors themselves, may suffer from emotional distress and frustration because of the misdiagnosis [7]. The delay in correct diagnosing postpones the prescription of DMT, which is supposed to decrease the MS burden in any particular patient. Thus, diagnostic delay and error in the primary diagnosis of MS may negatively affect the course of the disease. On the other hand, diagnostic mistakes may reflect an intrinsically more benign initial course of the disease, as it is, for instance, for ALS [6,8]. A PubMed search performed on 24 November 2019 using different combinations of keywords “multiple sclerosis”, “misdiagnosis”, “diagnostic errors”, “disability”, and “progression” yielded no studies, which would assess the influence of the diagnostic delay and initial MS misdiagnosis on the course of disease in MS patients.

Our aim, therefore, was to investigate the association between initial MD and reaching disability milestones in patients with RR-MS. 

## 2. Materials and Methods

In this retrospective cross-sectional study, clinical data from 128 RR-MS patients, receiving care at the Odesa Regional Medical Center for Mental Health between 2007 and 2018 was analyzed in accordance with the prespecified protocol. The data for the study was obtained from the center-based medical record database. Each individual entry to the study spreadsheets was coded, and no personal information that would allow the identification of a person was included. The local Ethics Committee approved the study protocol (ethical review of research protocol #6, approval date 09 October 2018). Collected data included the parameters that were identified by existing studies as the risk factors for disability progression in patients with RR-MS, such as patients’ sex, age of MS onset, smoking, DMT, BMI [1], along with years before the sustained EDSS 4 and 6 from MS onset, prehistory of an initial misdiagnosis, specification of the incorrect diagnosis and the neurologic symptoms the patients had at the first presentation to a physician. The first presentation may have happened in other centers. EDSS 4 and EDSS 6 were designated as disability milestones due to the association with a significant decrease in ambulation. EDSS 4 is the score at which the distance patient can make do without support but is beginning to be considered, and EDSS 6 is the score at which at least unilateral assistance is needed to walk 100 meters. Statistical analysis was performed using R (version 3.5.1) libraries and packages. For all applicable methods, the two-tailed analysis was used and *p* < 0.05 was considered significant. Continuous variables were assessed for normality (graphical visualization and *D’Agostino* K2 normality test) and processed using mean and standard deviation or median and interquartile range as appropriate. Categorical variables were described using frequencies. Univariate analysis with the Kaplan–Meier method was used to investigate the effect of initial misdiagnosis on reaching EDSS 4 and EDSS 6. A Cox proportional-hazards model with initial misdiagnosis and sex, history of smoking, patients’ age, age of MS onset, BMI, and DMT as independent variables was used to identify whether the consideration of a combination of these clinical parameters affects reaching the defined milestones. Multivariate logistic regression with initial misdiagnosis as the dependent variable and the symptoms the patients had at the first presentation to a physician as independent variables were used to study the association of the symptoms the patients with presumable MS had at the first presentation to a physician with the initial misdiagnosis. 

## 3. Results

### 3.1. Studied Population

Study population included 84 female (65.6%) and 44 male (34.4%) patients. The mean age of patients was 37.8 ± 10.7 years (females 38.7 ± 11 years, males 36 ± 9.7 years), mean age of onset was 29.7 ± 9.6 years (females 30.6 ± 10.2 years, males 27.1 ± 8.2 years). A total of 45.3% of all the patients received DMT. Male and female groups did not have statistically significant differences in terms of characteristics except for smoking (27.3% in male group and 11.9% in female group) and DMT (40.9% in male group and 47.6% in female group). Other characteristics of the population are shown in Table 1.

### 3.2. Impact of Misdiagnosis on Disease Progression

**Figure 1:**
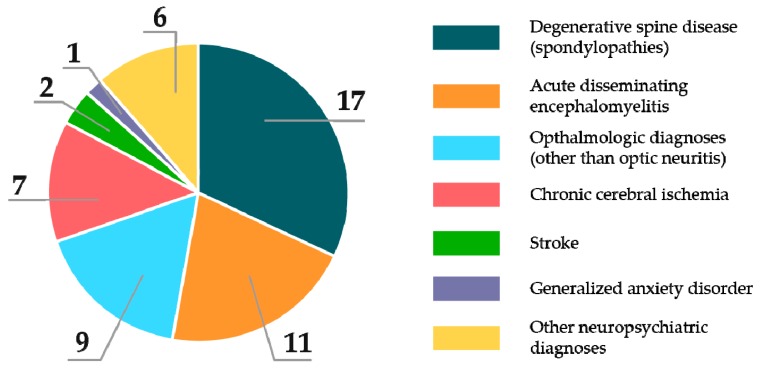
Conditions patients with relapsing-remitting (RR) multiple sclerosis (MS) were initially misdiagnosed with.

Degenerative spine disease (spondylopathies) accounted for the majority of alternative diagnoses on primary referral (17 cases) followed by acute disseminated encephalomyelitis (11 cases), other ophthalmologic pathologies but optic neuritis (9 cases), chronic cerebral ischemia (7 cases), stroke (2 cases), generalized anxiety disorder (1 case), as well as other neuropsychiatric disorders (6 cases), as shown in Figure 1. A larger proportion of males was misdiagnosed compared to females, but the difference was statistically insignificant.

**Figure 2:**
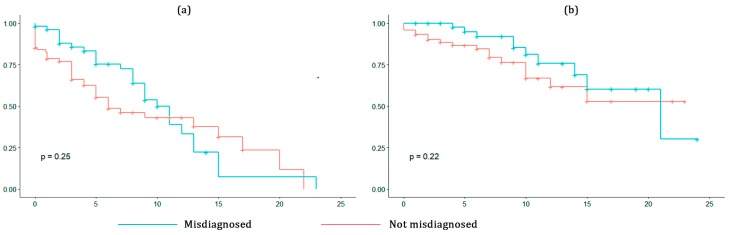
(**a**) Kaplan–Meier curves for reaching EDSS 4 in misdiagnosed and not misdiagnosed patients; (**b**) Kaplan–Meier curves for reaching EDSS 6 in misdiagnosed and not misdiagnosed patients.

Patients misdiagnosed at the disease onset seemed to have a lesser risk to acquire EDSS 4 up to 11 years from the onset. After that, misdiagnosed patients tended to decline faster than non-misdiagnosed, but the result was statistically insignificant (Figure 2a). Median time to acquire EDSS 4 in misdiagnosed patients was eight years (95% CI 0.0–17.6), in non-misdiagnosed—three years (95% CI 0.0–20.0 years).

For EDSS 6 (Figure 2b), misdiagnosed patients showed a lesser risk of progression up to 22 years of disease and a higher risk after this time point (*p* = 0.22). Median time to acquire EDSS 6 was 10 years (95% CI 4.25–20.75) in misdiagnosed patients and five years (95% CI 0.0–13.73) in non-misdiagnosed.

In a multivariate Cox proportional-hazards model with initial misdiagnosis and sex, history of smoking, patients’ age, age of MS onset, BMI, and DMT as independent variables, initially misdiagnosed patients showed a statistically insignificant trend towards a lesser chance of reaching both EDSS 4 (HR 0.81, 95% CI 0.46–1.42) and EDSS 6 (HR 0.55, 95% CI 0.24–1.3).

Among statistically significant predictors, male sex was associated with a lower hazard (HR 0.49, 95% CI 0.26–0.92, *p* = 0.03), and age at onset of more than 50 years was associated with a higher hazard (HR 4.01, 95% CI 1.23–13.10, *p* = 0.02) of reaching EDSS 4 (Table 2). There were no statistically significant predictors of reaching EDSS 6 (Table 3).

### 3.3. Symptoms at Onset as Predictors of Misdiagnosis

Multiple regression analysis could not identify symptoms at the initial encounter, which would be associated with the initial misdiagnosis (Table 4). Instead, ataxia was statistically significantly associated with lesser odds of incorrect diagnosis at the initial presentation (OR 0.28, 95% CI 0.23–0.85, *p* = 0.03).

## 4. Discussion

In our study, there was no clear evidence that misdiagnosis at the disease onset influences reaching disability milestones in patients with RR-MS. There was a trend for the initial misdiagnosis in patients with RR-MS to be associated with a lesser risk of reaching disability milestones compared to patients who were diagnosed correctly at the first encounter. However, the result was not statistically significant, neither if analyzed alone, nor adjusted for the additional factors, such as patients’ sex, age, BMI, age of MS onset, smoking, and DMT. 

On the other hand, chronic conditions with a fluctuating progressive course are often misdiagnosed due to the nonspecific clinical picture. ALS is an example of such a disorder, in which initial misdiagnosis was shown to be associated with longer survival [6,8]. We could not identify any studies assessing the association between initial misdiagnosis and MS course, so for our study, we supposed that in MS, this association could follow either general or ALS scenario. Although not reaching statistical significance, the association showed a trend to follow the ALS scenario, i.e., lesser risk of reaching disability milestones in initially misdiagnosed patients. 

This association might reflect intrinsically a more benign condition resulting in mild or nonspecific symptoms present at the onset preventing and slowing the progression. 

As an additional finding from the performed analysis, in the Cox proportional-hazards model for reaching EDSS 4 that considered initial misdiagnosis, sex, smoking, age at MS onset, BMI, age of the patients, and DMT as independent variables, male sex was found to decrease the risk of reaching EDSS 4 (HR 0.49, 95% CI 0.26–0.92, *p* = 0.027). Findings of existing studies show a faster disability progression in male patients [1]. This discrepancy might be attributed to the fact that different studies use different milestones to define disability. This assumption is supported by the fact that in our study, the mentioned association between male sex and lesser hazards of reaching the disability milestone was not reproduced in the same analysis for EDSS 6. Another statistically significant predictor of reaching EDSS 4 was the age of MS onset, but this time, the findings aligned with the results of other studies indicating that the older age at the MS onset is associated with more chances of reaching disability, although the range of data distribution was high (HR 4.01, 95% CI 1.23–13.10, *p* = 0.021).

We could not draft the clinical picture of a patient that would be at the highest risk of misdiagnosis, but we could identify ataxia as a symptom associated with a lesser chance of misdiagnosis. Cerebellar dysfunction that was identified as a hallmark of MS back in the second half of the 19th century by Jean Martin Charcot seems to guide the clinical diagnosis of MS today as well.

Our study has numerous limitations. This is a retrospective study based on the medical records, and the way the physician-patient encounters are recorded might impact the quality of the obtained data. The prospective design of such a study with standardized recording would eliminate this limitation. We also did not analyze the delay in the first physician encounter since the beginning of the symptoms and DMT administration to the misdiagnosed vs. non-misdiagnosed patients, which might also impact the rate of reaching disability milestones.

The results of the study should be interpreted as an appeal to be attentive to any subtle but suspicious of MS presentations because, in MS patients with mild symptoms, there is a larger field for disability prevention with DMT than in those with moderate or severe symptoms. 

## 5. Conclusions

From the obtained results, there is no clear evidence that initial misdiagnosis affects reaching DMs in MS patients, although there is a trend of a lesser chance of reaching disability milestones in misdiagnosed patients. Caution should be taken not to miss the patients presenting with a vague clinical picture for a timely establishment of the diagnosis and initiation of DMT.

## Figures and Tables

**Table 1:** Characteristics of the study population.

Characteristics	Total (N = 128)	Females (N = 84)	Males (N = 44)
Age	37.8 ± 10.7	38.7 ± 11	36 ± 9.7
Onset age	29.7 ± 9.6	30.6 ± 10.2	27.1 ± 8.2
Smoking ^1^	22 (17.2%)	10 (11.9%)	12 (27.3%)
Received DMT ^1^	58 (45.3%)	40 (47.6%)	18 (40.9%)
BMI	23 ± 3.8	22.9 ± 3.8	23.4 ± 3.8
EDSS at onset ^2^	2.5 (1.5–3)	3.0 (1.5–3.0)	2.0 (1.0–3.0)
Follow-up (years) ^2^	7 (3.0–11.25)	7 (3.0–10.25)	8.5 (3.0–12.25)
Reached EDSS 4	67 (52.3%)	50 (59.5%)	17 (38.6%)
Reached EDSS 6	29 (22.7%)	21 (25%)	8 (18.2%)

^1^—features for which *p* < 0.05, ^2^—median (interquartile range).

**Table 2:** Hazard ratios (HR) and 95% confidence intervals (CI) of the association between initial misdiagnosis, sex, smoking, age at MS onset, BMI, age of the patients, and disease-modifying therapy (DMT) and reaching EDSS 4.

Predictors of Reaching the Milestone	HR	CI (95%)	*p*
Misdiagnosis			
- Not misdiagnosed (N = 75)	-	-	1
- Misdiagnosed (N = 53)	0.81	0.46–1.42	0.46
Sex			
- Female (N = 84)	-	-	1
- Male (N = 44)	0.49	0.26–0.92	0.03
History of smoking			
- No (N = 106)	-	-	1
- Yes (N = 22)	1.41	0.71–2.82	0.33
Age at MS onset			
- <18 (N = 9)	0.44	0.12–1.55	0.2
- 18–50 (N = 114)	-	-	1
- >50 (N = 5)	4.01	1.23–13.10	0.02
BMI			
- <18.5 (N = 8)	1.87	0.79–4.46	0.16
- 18.5–24.9 (N = 88)	-	-	1
- 25–29.9 (N = 22)	1.20	0.58–2.46	0.63
- ≥30 (N = 10)	0.99	0.37–2.65	0.98
Age			
- 18–50 (N = 107)	-	-	1
- >50	0.99	0.47–2.07	0.98
DMT			
- Did not receive DMT (N = 70)	-	-	1
- Received DMT (N = 58)	0.75	0.45–1.26	0.28

**Table 3:** Hazard ratios (HR) and 95% confidence intervals (CI) of the association between initial misdiagnosis, sex, smoking, age at MS onset, BMI, age of the patients, and DMT and reaching EDSS 6.

Predictors of Reaching the Milestone	HR	CI (95%)	*p*
Misdiagnosis			
- Not misdiagnosed (N = 75)	-	-	1
- Misdiagnosed (N = 53)	0.55	0.24–1.3	0.17
Sex			
- Female (N = 84)	-	-	1
- Male (N = 44)	0.87	0.35–2.2	0.77
History of smoking			
- No (N = 106)	-	-	1
- Yes (N = 22)	1.15	0.39–3.3	0.8
Age at MS onset			
- <18 (N = 9)	0.61	0.07–4.9	0.64
- 18–50 (N = 114)	-	-	1
- >50 (N = 5)	2.49	0.39–15.8	0.33
BMI			
- <18.5 (N = 8)	1.92	0.53–6.9	0.318
- 18.5–24.9 (N = 88)	-	-	1
- 25–29.9 (N = 22)	2.2	0.82–5.9	0.12
- ≥30 (N = 10)	1.04	0.22–4.9	0.96
Age			
- 18–50 (N = 107)	-	-	1
- >50	1.34	0.49–3.7	0.57
DMT			
- Did not receive DMT (N = 70)	-	-	1
- Received DMT (N = 58)	0.66	0.3–1.4	0.29

**Table 4:** Impact of symptoms at the initial presentation of the RR-MS patients on misdiagnosis (* *p* < 0.05).

Clinical Symptoms	β	Standard Error	Wald’s χ^2^	OR	CI (95%)	*p*
Ataxia	−1.27	0.58	4.8	0.28	0.23–0.85	0.03 *
Impaired eye movements	−0.87	0.67	1.7	0.41	0.1–1.45	0.19
Optic neuritis	−0.94	0.51	3.4	0.38	0.14–1.02	0.06
Sensory loss	−0.74	0.46	2.6	0.48	0.18–1.16	0.11
Pyramidal dysfunction	0.39	0.42	0.89	1.48	0.66–3.34	0.35
Bulbar symptoms	−1.47	0.88	2.8	0.23	0.03–1.13	0.09
Pelvic organs dysfunction	−0.79	0.91	0.75	0.44	0.17–1.88	0.39
